# Better management of multimorbidity: a critical look at the ‘Ariadne principles’

**DOI:** 10.1186/s12916-014-0222-2

**Published:** 2014-12-08

**Authors:** Peter Bower

**Affiliations:** NIHR School for Primary Care Research, Manchester Academic Health Science Centre, University of Manchester, Manchester, UK

**Keywords:** Assessment, Multimorbidity, Treatment burden, Treatment goals

## Abstract

Primary care clinicians and researchers are growing increasingly aware of the prevalence of multimorbidity among long-term conditions, and the impact on patient experience, health, and utilisation of care. The correspondence paper by Muth et al. entitled ‘*The Ariadne principles: how to handle multimorbidity in primary care consultations*’ outlines new thinking on a better way to manage the challenges of decision-making in multimorbidity. The paper highlights the importance of shared treatment goals as a fundamental basis for more effective management. Although a welcome contribution to the literature, the principles raise a number of challenges: the complexities of achieving effective patient-centred assessment and goal-setting; how best to encourage implementation of new practices; and the current state of the evidence around multimorbidity and its management.

Please see related article: http://www.biomedcentral.com/1741-7015/12/223.

## Background

Despite the gains in quality of care associated with clinical guidelines and other improvement initiatives, there is a growing perception that the current direction of primary care has significant downsides and that these may be magnified in the context of patients with multimorbidity [1].

The focus on care for single conditions has undoubtedly improved aspects of care [2], but application of single condition guidelines means that multimorbid patients face significant burden of treatment: multiple appointments, competing demands for self-management, and challenges in navigating care [3,4]. Equally, practitioners struggle to make sense of multiple guidelines, to prioritise interventions, and to co-ordinate their activity with other professionals [5,6].

The Ariadne principles outlined by Muth et al. represent an ambitious attempt to reorient primary care back towards a more patient-centred vision [7]. These are not formal guidelines, but a list of core principles that could guide care delivery in the context of multimorbidity, and chime with some of the issues highlighted in a recent critique of evidence-based medicine [8].

## Discussion

The principles may cause conflicting responses in some readers. One likely response is that they simply represent what would be expected of any ‘good GP’ , harking back to well-known principles of ‘patient centred’ care which have been outlined before [9-11]. Although there are important modifications to take account of multimorbidity, the essence may well be familiar.

The opposite reaction may be that the principles are face valid, but that achieving these principles with patients in the reality of busy clinics, with limited resources, and with patients already struggling under multiple pressures, represents a standard which is unlikely to be achieved routinely in practice.

The Quality and Outcomes Framework (QOF) is a popular whipping boy as a potential cause of the single-condition, guideline-focussed care which is seen as so problematic in the context of multimorbidity. Martin Roland is fond of challenging critics to remember the major gaps in quality that existed before QOF and other initiatives that improved the care of single long-term conditions. Other readers may be reminded of an earlier study of patient-centred training, which saw more satisfied patients, but also reductions in clinical quality measures [12]. Would the introduction of the Ariadne principles in the context of the QOF lead to similar tensions?

If the Ariadne principles can be married to effective clinical care, it is worth highlighting three additional challenges.

A core part of Ariadne is an improved assessment, leading to a fuller discussion about patient goals, and a care plan to assist in achieving those goals. Although perfectly reasonable in principle and in line with thinking in behaviour change, the authors mention a wide range of factors that need to be included in this assessment, including social circumstances, financial constraints, living conditions and social support, health literacy, functional autonomy and coping strategies. All very sensible, but how are these to be assessed in a way that is reliable and useful, and how can those factors be used to deliver better care? The experience of depression assessments in the UK found significant professional resistance to standardised measurement [13]. Of course, it is argued that GPs are in an excellent position to know their patients, and that a return to professional judgement and clinical decision-making is needed: Greenhalgh et al. talk of ‘*rapid, intuitive reasoning informed by imagination, common sense, and judiciously selected research evidence and other rules*’ [8]. However, GP assessments of important facets of their patients are not always accurate [14,15]. How can we ensure equity and consistency in how these complicated factors are assessed and somehow ‘*taken into account*’ [16] in clinical decision-making?

In support of Ariadne, there is a fair amount of agreement on the broad nature of clinical practice best suited to managing multimorbidity. The bigger challenge may be around implementation. Many primary care interventions aimed at changing practitioner behaviour have fallen foul of the adage that feasible changes are not effective, and effective changes are not feasible. Financial incentives may be poorly suited to facilitating these principles, as they are a blunt tool and unlikely to lead to high quality implementation of complex behaviours – the experience of the depression incentives being a case in point [17]. There is also an important issue around patient preparation. Thinking about priorities among treatments and outcomes, setting SMART goals, becoming ‘activated’ – all these may be as unfamiliar to some patients as they are to practitioners.

Finally, the evidence base concerning multimorbidity is relatively limited [18,19], and it may be premature to suggest fundamental changes to care on the basis of limited data. There is a significant qualitative literature on the problems faced by patients and practitioners in managing multimorbidity [3,20,21], but the quantitative literature is much thinner. Our own study comparing the experience of chronic illness care in patients with single and multiple conditions found few differences [22]. Although the experiences reported in qualitative research are undoubtedly salient and valid, those experiences may not be universal. Intervening to reduce treatment burden on patients will only lead to major improvements if burden is a key driver of poor outcomes, and we are only beginning to understand the impacts and how they might be reduced.

The potential of principles such as Ariadne is theoretically profound, but caution is required before assuming that these benefits will be achieved. Large scale, pragmatic evaluations of the delivery and impact of these new models of care are required and, fortunately, such evaluations are now underway [23].

## Conclusions

The Ariadne label, derived from the myth of Theseus and the Minotaur, is a lovely analogy for these multimorbidity principles, as Ariadne used a ball of twine to help her lover navigate out of a complex situation to freedom. However, that story did not have a happy ending for Ariadne. Avoiding a similar fate for her principles will require attention to the challenges that exist and a hard-headed assessment of their impact on care outcomes.
